# Artificial Metalloproteins: At the Interface between Biology and Chemistry

**DOI:** 10.1021/jacsau.2c00102

**Published:** 2022-06-02

**Authors:** Spencer A. Kerns, Ankita Biswas, Natalie M. Minnetian, A. S. Borovik

**Affiliations:** Department of Chemistry, University of California, 1102 Natural Science II, Irvine, California 92797, United States

**Keywords:** Artificial Metalloproteins, Active Sites, Catalysis, Structural Biology

## Abstract

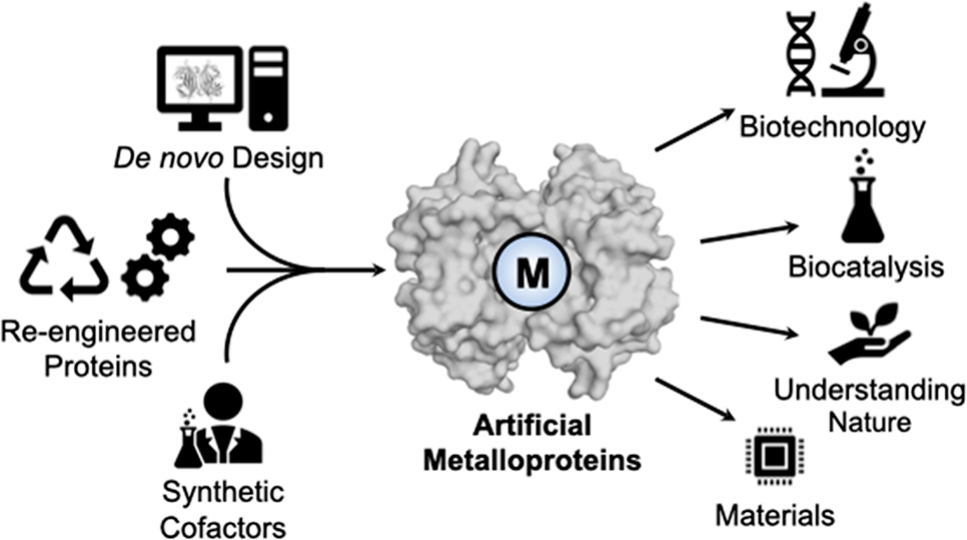

Artificial metalloproteins (ArMs) have recently gained significant interest due to their potential to address issues in a broad scope of applications, including biocatalysis, biotechnology, protein assembly, and model chemistry. ArMs are assembled by the incorporation of a non-native metallocofactor into a protein scaffold. This can be achieved by a number of methods that apply tools of chemical biology, computational *de novo* design, and synthetic chemistry. In this Perspective, we highlight select systems in the hope of demonstrating the breadth of ArM design strategies and applications and emphasize how these systems address problems that are otherwise difficult to do so with strictly biochemical or synthetic approaches.

## Introduction

Metalloproteins constitute an estimated one-third to half of the proteome.^1^ It is now recognized that metal ions play essential roles in governing the structural features and catalytic function of many proteins necessary for proper physiological function.^2^ Our understanding of the specific roles of metal ions within proteins has been advanced by synergistic efforts in chemical biology and synthetic chemistry to investigate the underlying structure–function relationships. Fundamental insights from structural biology, biophysics, and the development of truncated small-molecule synthetic mimics of metalloprotein active sites have informed our understanding that both the primary coordination sphere of the metal ion and the surrounding protein microenvironment, or secondary coordination sphere, are essential for proper function. Proteins with artificially engineered metal active sites is one approach that has produced systems that has informed us on the subtle connections between structure and function. The advancement of synthetic methodologies and biochemical tools such as protein engineering has recently reinvigorated the study of artificial metalloproteins (ArMs). This hybrid approach at the interface of chemical biology and synthetic chemistry aims to prepare new systems that address problems in a wide range of applications, including biocatalysis,^3^ biotechnology,^4,5^ protein structure and assembly,^6,7^ and model chemistry.^8^

**Figure 1:**
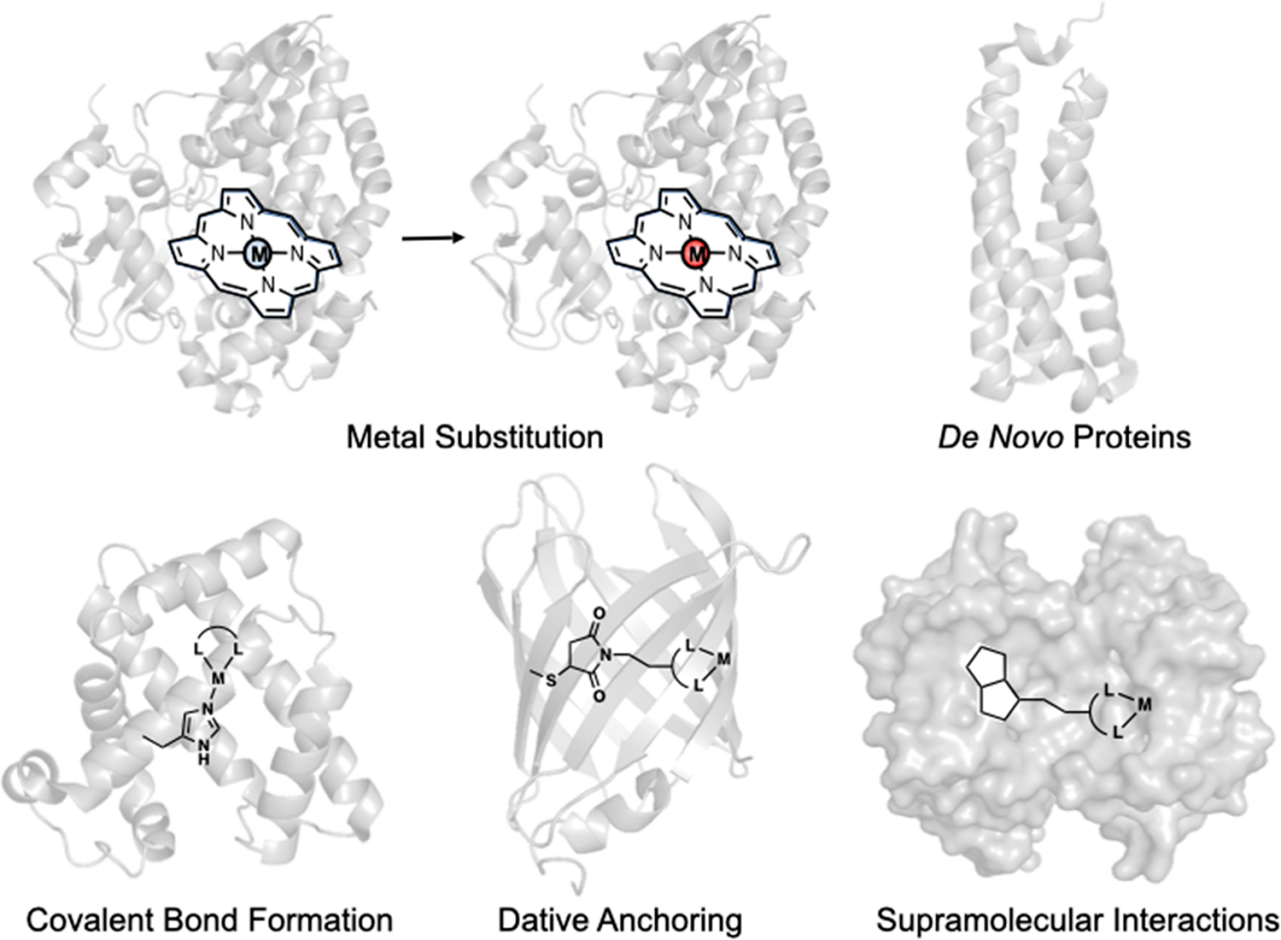
Schematic representations of different methods employed to construct artificial metalloproteins (ArMs).

Artificial metalloproteins are constructed by the introduction of non-native metallocofactors into a naturally occurring protein or through the design of *de novo* protein matrices, as summarized in Figure 1. Insertion of the metallocofactor can be accomplished by the reconstitution of an apoprotein (either native or re-engineered) with a non-native metal or the addition of an artificial synthetic construct to the protein.^9^ Synthetic metal complexes can be incorporated by a variety of means, including (i) formation of a direct dative, covalent bond to an amino acid ligand, (ii) covalent attachment to the protein by a bioconjugate moiety, or (iii) noncovalent anchoring in the protein environment. Thus, artificial metalloproteins offer the opportunity to incorporate both nonbiologically relevant metals *and* designed ligands in a platform that also benefits from the aforementioned mutagenesis of proteins. Here, we present selected examples from the literature that survey the different approaches to prepare ArMs. In this Perspective, we have focused on the choice of protein host in a manner that highlights how both native metalloproteins and proteins that do not contain native metal sites can be applied to the design of artificial metalloproteins.

## Re-engineering Metalloproteins

One of the most obvious approaches for constructing ArMs is to re-engineer a metalloprotein to produce new active sites with structural and functional properties that differ from those of the native protein.^10−20^ Some of the best examples of this approach are found in heme metalloenzymes, which are a versatile class of proteins with functions ranging from reversible dioxygen binding to C–H bond functionalization. Many re-engineering studies have left the heme cofactor intact and have focused on determining the contributions of residues within the active site toward functions. Using site-directed mutagenesis methods, single point mutations are made to change either the axial ligands at the Fe centers or those around the Fe housed within the secondary coordination sphere. In addition, the seminal work of Arnold on reengineered cytochrome P450s (P450s) using directed evolution methods has strongly affected a variety of different fields, including approaches relevant to ArMs.^21−26^ Hartwig has also shown that the substitution of Fe porphyrins with unnatural metalloporphyrins within heme proteins produces an array of ArMs with new functions.^15,16,27,28^ There are a number of reviews that have already discussed the importance of these studies, and they will not be reiterated here;^29,30^ instead, we will describe the utility of using heme metalloproteins to engineer additional metal ion binding sites to produce new metallocofactors.

### Artificial Heme-Copper Oxidases

**Figure 2:**
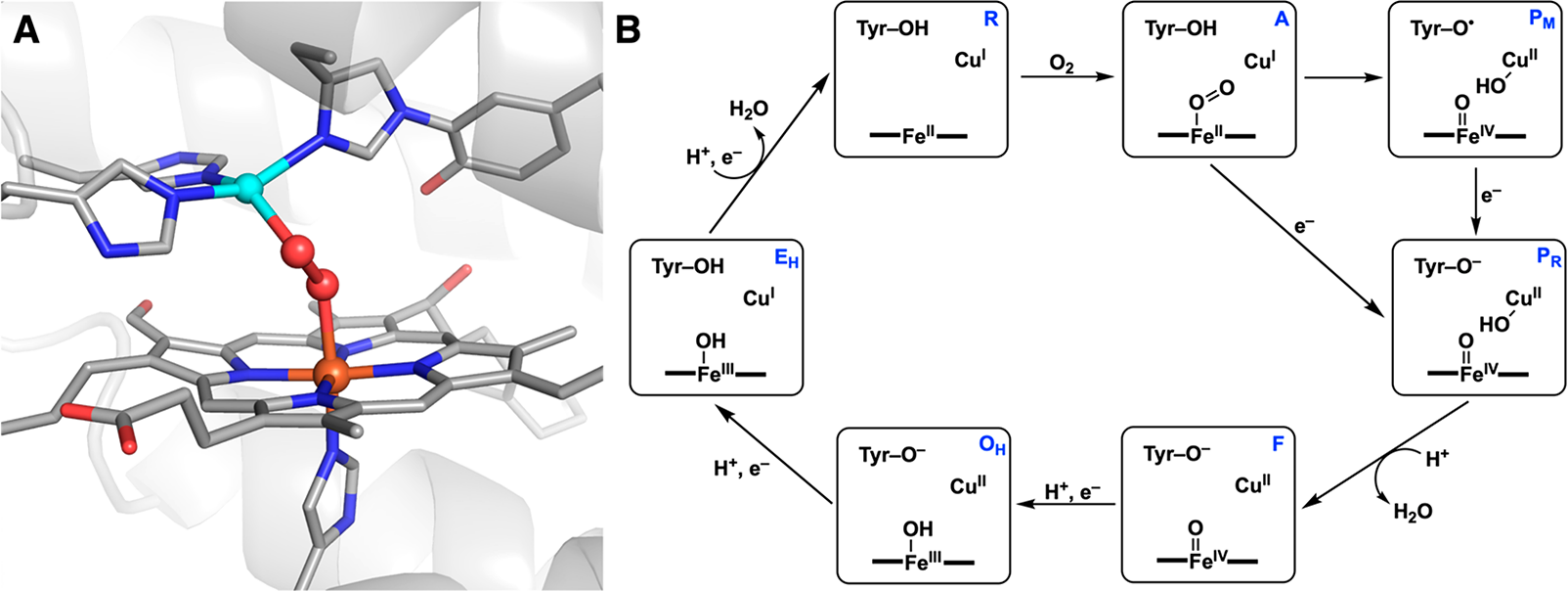
Peroxide-bound heme-Cu_B_ active site of bovine heart cytochrome *c* oxidase (PDB: 2OCC), a member of the HCO family (A). Schematic representation of the mechanism of O_2_ reduction in cytochrome *c* oxidases (B).

Bimetallic active sites that include one heme unit are well-known, with one prominent example being the heme-copper oxidases (HCOs)—these metalloproteins are a family of terminal oxidases that are part of the respiratory pathways of eukaryotic mitochondria and bacteria. Their function is to reduce O_2_ to water, using the energy from that reaction to generate a transmembrane proton gradient which ultimately leads to ATP synthesis. The site of binding and subsequent reduction of O_2_ is the heme-Cu_B_ bimetallic center (Figure 2).^31−33^ Spectroscopic signals from native HCOs are often masked by the signal from other low-spin heme centers, and difference spectra have to be used to study the heme-Cu_B_ center. To circumvent these problems, engineering ArMs has been successful in recreating just the heme-Cu_B_ bimetallic center in a protein scaffold and determining the role of the Cu_B_ site in O_2_ binding and reduction through spectroscopy. We recognize that there are now many synthetic inorganic models that mimic these bimetallic interactions;^34,35^ however, re-engineered ArMs from existing heme proteins have the advantage of using the same type of ligands that are found in the native HCOs. Moreover, they can be examined under the same physiological conditions.

A redesigned cytochrome *c* peroxidase with a Cu binding site near the heme center (Cu_B_C*c*P) was the first protein model of the heme-Cu_B_ site found in cytochrome *c* oxidases that exhibited spin coupling between the Cu(II) and the heme-Fe(III) center without any addition of exogenous ligands.^36^ Later Lu redesigned sperm whale myoglobin with a Cu binding site near the heme center (denoted Cu_B_-Mb, which contains the heme site and a proximal binding site without coordinated metal ions). Cu_B_-Mb has a low affinity for O_2_ in the absence of any metal ions in the Cu binding site, but increased affinity for O_2_ was found in the presence of Ag(I) ions; however, this ArM was unable to further reduce dioxygen.^37^ In contrast, when Cu_B_-Mb is treated with Cu(II) ions and a reductant, binding and reduction of dioxygen was observed. These findings suggest that the Cu center is structurally important for efficient O_2_ binding to the heme center in HCOs and is essential for reduction.

The ArM Cu_B_-Mb reduced O_2_ in the presence of Cu(II), and reductants formed a peroxy-heme intermediate that subsequently underwent heme degradation to verdoheme—a reaction observed in heme oxygenases (HOs).^38^ This contrasts with the formation of high-valent Fe(IV)–-oxido intermediates that are observed in HCOs and P450 enzymes. Biochemical studies conducted on P450s suggest that a hydrogen-bonding network of water molecules and hydrophilic residues in the distal pocket of the heme protein supplies protons to the peroxy-heme and facilitates conversion to the reactive Fe(IV)–oxido species. Lu and co-workers therefore proposed that Cu_B_-Mb may lack a similar H-bonding network (and proton delivery mechanism), which may explain why Cu_B_-Mb does not exhibit HCO activity. To test this hypothesis, H_2_O_2_ was added to the Fe(III)/Cu(II) Cu_B_-Mb form (met-Cu_B_-Mb), which resulted in the formation of an Fe(IV)–oxido species. They proposed that the additional protons liberated upon coordination of H_2_O_2_ facilitated formation of the Fe(IV)–oxido species, similar to the role of the hydrogen-bonding network in P450s, and suggested that similar hydrogen-bonding networks may be important to HCO function. With this work, Lu and co-workers have demonstrated the power of their approach by transforming an O_2_ carrier protein such as myoglobin and engineering it to display both HO and HCO activity in a manner that was informative of the probable mechanisms of native HCO enzymes.

### Artificial Non-Heme Hydroxylases

Artificial metalloproteins have also been prepared using native metalloprotein hosts and synthetic metallocofactors to produce new active sites; this approach is in contrast to reproducing the native active site that was discussed above. Marchi-Delapierre, Cavazza, and Ménage utilized this approach to develop an ArM capable of dioxygen binding, activation, and arene hydroxylation.^39^ In this system, the advantages of both synthetic chemistry and protein chemistry were leveraged in order to visualize, through XRD methods, reactive intermediates over the course of the catalytic reaction. The high solvent content and molecular flexibility endogenous to protein crystals enables the possibility to trap and structurally characterize reactive intermediates during catalysis. However, due to the highly evolved nature of enzyme active sites, catalytic reactions occur very rapidly, which often makes it difficult to trap reactive intermediates. Catalytic transformations by small molecules typically proceed at much slower rates, but similar *in crystallo* reactions are challenging to perform because of their more rigid crystal lattices. To address these obstacles, Ménage et al. have successfully engineered a system that incorporates a small-molecule catalyst into a protein host to structurally characterize various catalytic intermediates.

**Figure 3:**
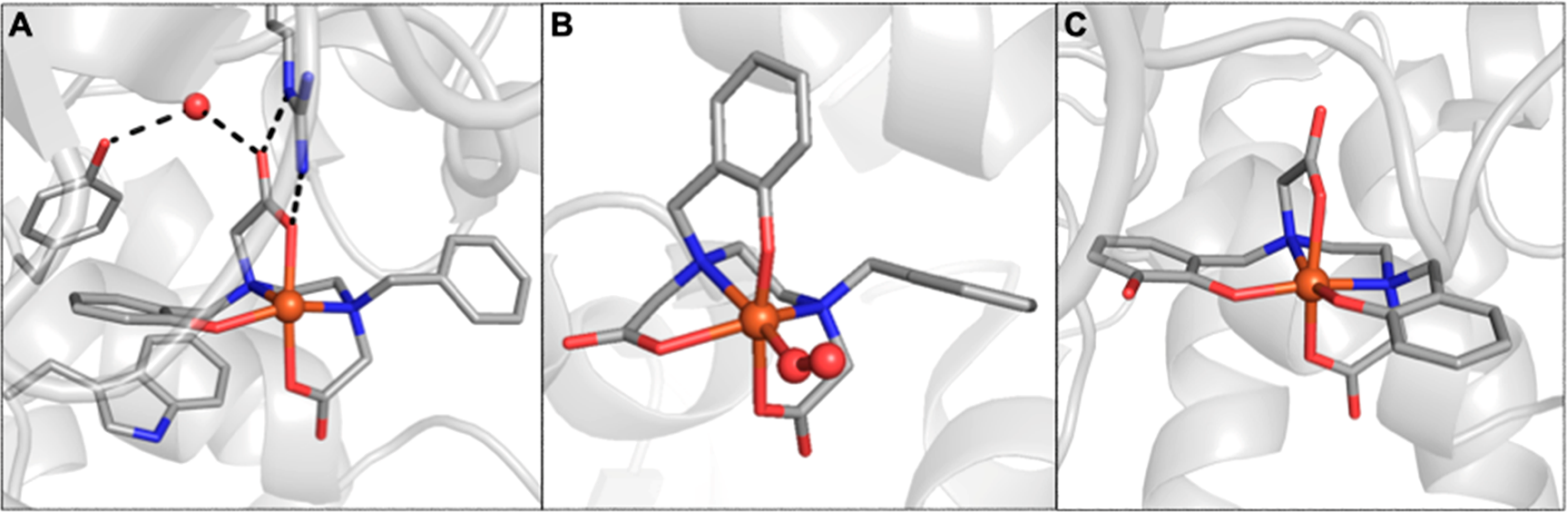
Structures of an artificial Fe hydroxylase depicting the noncovalent interactions immobilizing the [Fe(L)OH]^−^ cofactor in *NikA* (A) (PDB: 3MVW), the captured dioxygen bound intermediate species (B) (PDB: 3MVY), and the doubly hydroxylated product resulting from intramolecular hydroxylation of the ligand (C) (PDB: 3MW0).

The synthetic iron complex [Fe(L)OH]^−^ (L = *N*-benzyl-*N*′-(2-hydroxybenzyl)-*N*,*N*′-ethyldiamine diacetate) was incorporated into the nickel-binding protein *NikA* through a series of noncovalent intramolecular H-bonding interactions with the protein host (Figure 3A). The exposure of crystals to dioxygen enabled the characterization of several intermediates of an intramolecular ligand hydroxylation reaction that were trapped in different subunits of the ArM: these include a rare example of an Fe–O_2_ adduct (Figure 3B) and the hydroxylated product (Figure 3C). To support these assignments, the *in crystallo* structural studies were supplemented by resonance Raman measurements on crystals that indicated hydroxylation of the phenyl ring of L and the presence of a Fe–peroxido species.

**Figure 4:**
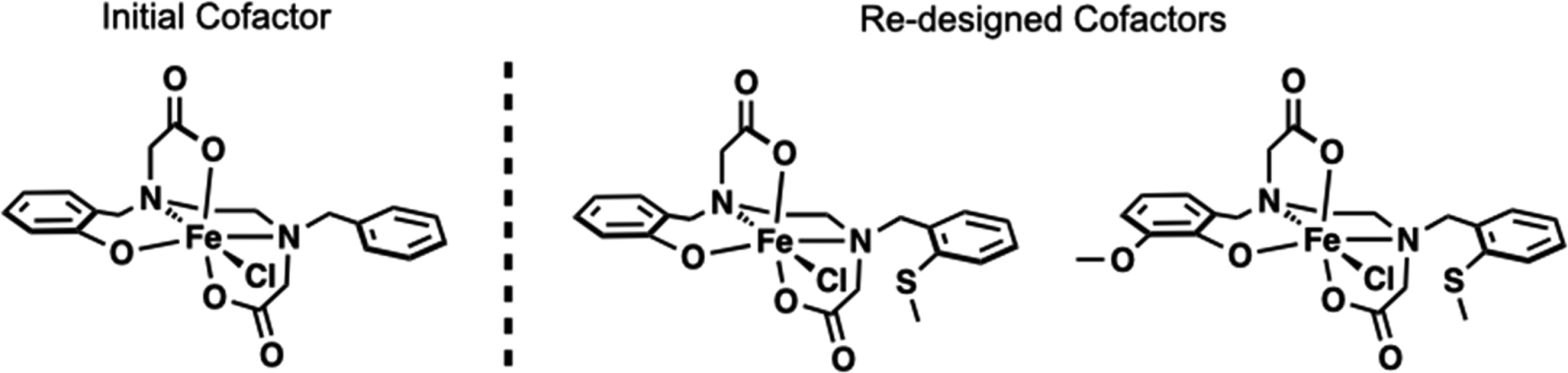
Artificial Fe cofactors used to prepare an artificial hydroxylase ArM capable of substrate oxidation.

More recently, Ménage and co-workers described how they were able to re-engineer the ArM crystals into heterogeneous catalysts that oxidize exogenous substrates.^40^ The active site was modified by redesigning the Fe complex used in the previous study via substituting functional groups at the reactive C–H bonds of the aromatic rings on L to prevent intramolecular hydroxylation (Figure 4). To increase the stability of crystals, they cross-linked the protein molecules within the lattice. This approach, denoted cross-linked enzyme crystals (CLEC), often produces more robust protein crystals that are stable under a variety of experimental conditions.^41−43^ For these artificial hydroxylases, the CLEC crystals were active heterogeneous catalysts in mixtures of water and organic solvents for months. Cross-linking the crystals also had important effects on function: it enabled both a wider scope of substrates and catalytic conditions to be explored than would typically be possible in a conventional biocatalytic investigation. After optimization of a range of oxidants, the engineered cross-linked hybrid catalyst proved to be a competent catalyst for the oxidation and hydroxychlorination of styrenyl substrates. When they are taken together, these studies offer an excellent demonstration of the ability to iteratively engineer ArMs that can trap reactive intermediates to determine the mechanisms to become highly functional catalysts.

## Reprocessed Metalloproteins

This approach also inserts an artificial metallocofactor into the active site of a native apo-metalloprotein; however, we distinguish this approach from those discussed above by the requirement that the structure of the artificial cofactor closely resembles that of the native metallocofactor and can then be used to establish structure–function correlations. The artificial cofactor has the advantage that it can be systematically modulated to examine how individual structural components effect the function. We highlight the benefits of this approach with a description of recent advances in hydrogenase chemistry.

### Artificial Hydrogenase Enzymes

**Figure 5:**
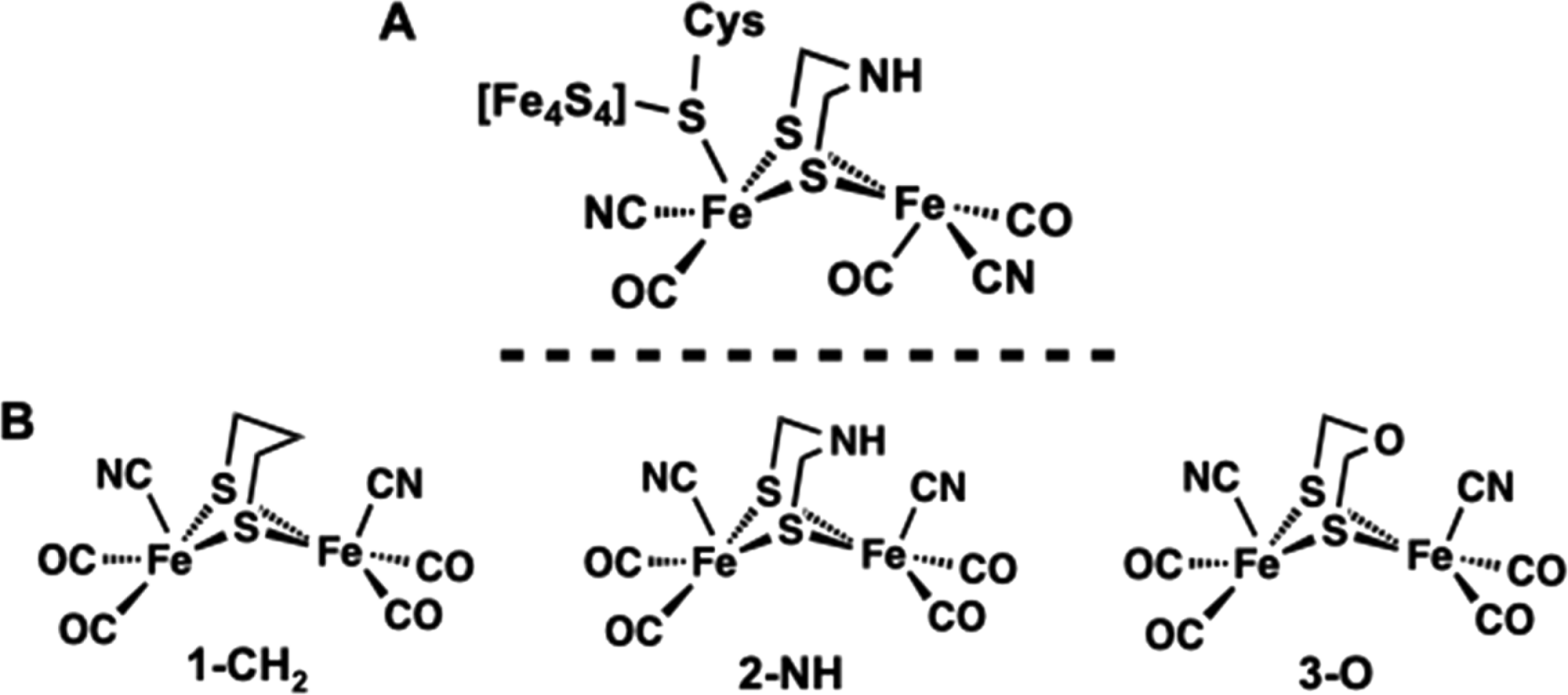
[2Fe] cofactor within the hydrogenase active site (A) and the synthetic complexes utilized to prepare artificial hydrogenases (B).

Hydrogenases are metalloenzymes found in microorganisms that catalyze both the reduction of protons to generate dihydrogen (H_2_) and the conversion of H_2_ to protons and electrons.^44^ There are three phylogenetically distinct classes of hydrogenase enzymes, which include the dinuclear [FeFe] and [NiFe] hydrogenases and a mononuclear [Fe] hydrogenase. The potential application of [FeFe] hydrogenases as a renewable source of H_2_ generation has inspired significant structural and mechanistic investigations by both biochemists and synthetic chemists. The active site of [FeFe] hydrogenase contains an asymmetric dinuclear [2Fe] core with each low-valent Fe(I) center coordinated by carbonyl (CO) and cyanide (CN^–^) ligands and a bridging dithiolate ligand (Figure 5A). While the use of CO and CN^–^ ligands is common in synthetic organometallic chemistry, it is rare to observe these ligands in nature due to the toxicity of these small molecules in the context of biology. Thus, the unique active site structure has inspired much curiosity about the biochemical machinery that produces the [2Fe] cofactor.

The use of ArMs constructed by combining synthetic metal complexes with hydrogenase maturase proteins has contributed to our understanding of the biosynthesis of the active site cofactor.^45^*In vitro* addition of synthetic Fe complexes at various stages of hydrogenase maturation has resulted in functional semisynthetic or artificial hydrogenase enzymes that demonstrates the efficacy of these complexes as surrogates of biochemical synthons.^46−48^ The use of these synthetic complexes and the ability to isotopically label the final [2Fe] cluster and specific ligands has allowed new advanced spectroscopic studies that enable the tracking of labeled species during the biosynthesis of the [2Fe] cofactor.^49,50^ To further illustrate how ArMs have provided insights to hydrogenase chemistry, we discuss studies on the development of functional artificial hydrogenases.

### Functional Artificial Hydrogenases and the Dithiolate Bridgehead

**Figure 6:**
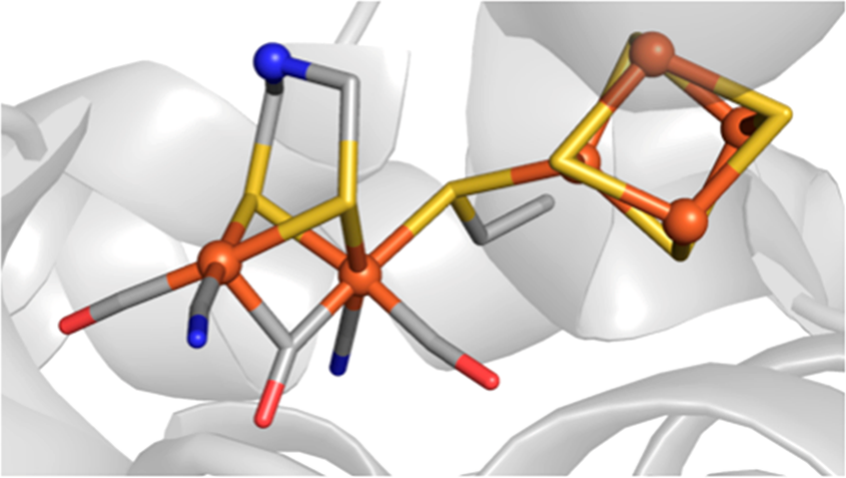
Structure of the artificial hydrogenase prepared with 2-NH and apo-CpI (PDB: 4XDC).

The X-ray crystal structure of a hydrogenase enzyme initially revealed that the bridging dithiolate moiety in the active site is connected by three light atoms; however, the identity of the bridging atoms could not be unambiguously assigned as a C, N, or O atom due to the resolution limitations of protein X-ray crystallography.^50−54^ Artero, Fontecave, Happe, and Lubitz demonstrated that a series of synthetic mimics could be incorporated into the hydrogenase apoprotein (*HydA*) without the need for the biological maturase proteins (i.e., *HydE*, *HydF*, and *HydG*).^55^ The synthetic complexes employed in the study depicted in Figure 5B represent the closest structural analogues to the endogenous active site and featured a variable bridging dithiolate moiety containing a C (1-CH_2_),^56−58^ N (2-NH),^59^ or O (3-O)^60^ atom at the bridgehead. *In vitro* addition of the synthetic complexes to the apoprotein produced artificial hydrogenases that feature a distinct atom (i.e., C, N, or O) at the bridgehead position of the thiolate ligand that is explicitly known because it was first synthesized and characterized outside of the protein. The hydrogen evolution activity revealed that only *HydA-2-NH*, featuring a N atom bridgehead, was capable of reproducing the activity of the native enzyme; *HydA-1*-CH_2_ and *HydA-3-O* did not demonstrate any H_2_ evolution. This finding provided key supporting evidence that [FeFe] hydrogenases likely contain an azadithiolate bridge that facilitates proton transfer during catalysis. The ability to leverage the atomistic control of synthetic chemistry and vary the identity of the bridgehead central atom would otherwise be difficult, if not impossible, to biosynthetically engineer, especially considering that the biosynthetic origin of this bridging moiety was previously unknown.^45^ Molecular structures of the artificial hydrogenases prepared with the apoprotein from *Clostridium pasteurianum* (CpI) were obtained by protein X-ray diffraction experiments^61^ (Figure 6) revealed that the identity of the bridgehead atom does not result in significant structural changes about the metallocofactors that may inhibit their activity. Thus, it was again concluded that the central amine is critical to proton transfer events that occur during catalysis. This work and the ability to reconstitute native enzymes from preformed and rigorously characterized synthetic complexes demonstrate one of the unique advantages of ArMs.

## Re-engineering Proteins into ArMs

**Figure 7:**
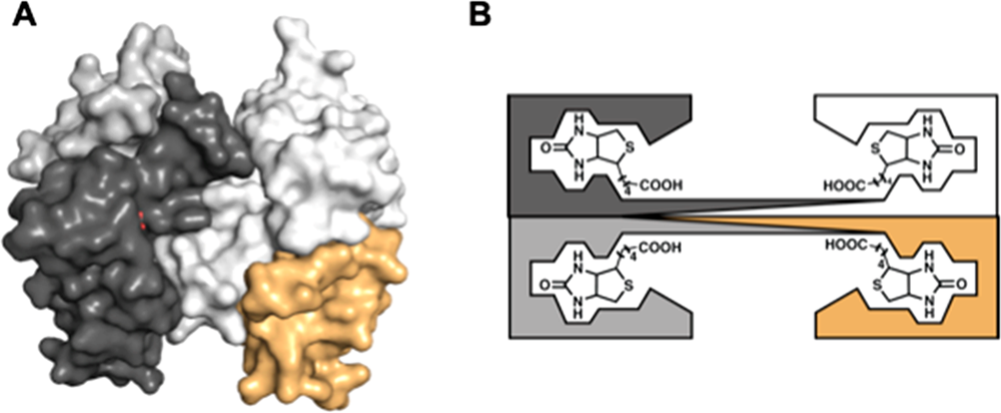
Surface structure of the tetrameric streptavidin (A). Schematic representation of the four monomers in streptavidin, each containing one biotin-binding site (B).

The above discussions all dealt with protein hosts whose natural functions required the binding of metal ions. A complementary approach is to confine artificial metallocofactors within proteins that have no prior capacity to bind metal ions.^62−68^ There are now several excellent examples of this approach, and we chose to feature ArMs prepared with biotin–streptavidin (Sav) technology. Streptavidin is a homotetrameric protein (Figure 7A) consisting of eight stranded β-barrels that assemble as a dimer of dimers, in which the biotin binding pockets face one another within a dimer (Figure 7B).^69^ The only known biological function of Sav is to bind its substrate biotin with exceptionally high affinity (*K*_d_ ≈ 10^–14^ M).^70,71^ Notably, Sav does not naturally bind a metallocofactor but artificial metalloproteins have been constructed using biotin–Sav technology by employing biotinylated ligands with metal-chelating moieties. Artificial metalloproteins of this type were first realized by Whitesides, who treated streptavidin (Sav) with a biotinylated organometallic Rh complex to produce an asymmetric hydrogenation catalyst.^72^

### Effects of the Secondary Coordination Sphere on Catalysis

**Figure 8:**
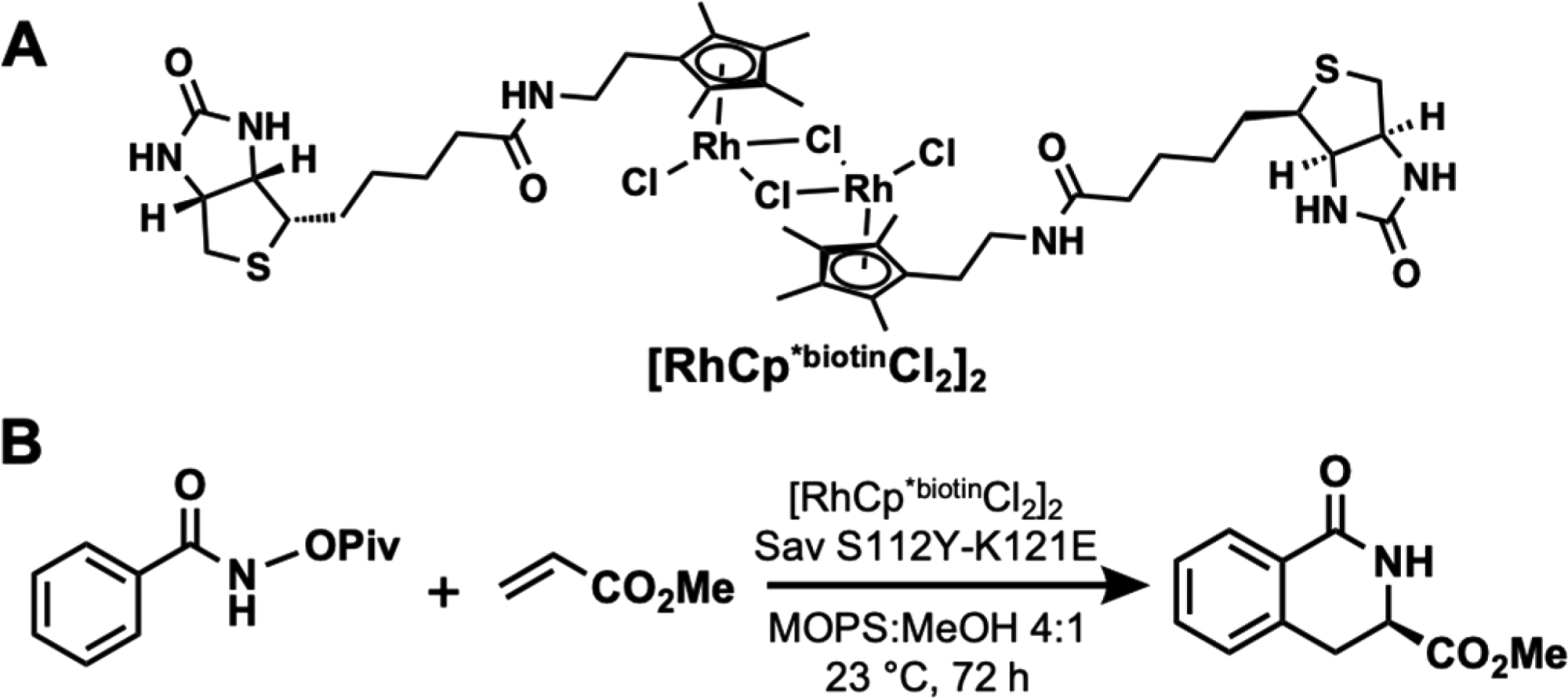
[RhCp^*biotin^Cl_2_]_2_ complex to construct an ArM using Sav (A) and the reaction catalyzed by the Rh ArM (B).

Ward has since greatly advanced the development of ArMs using Sav as the protein host, and he has reported on a variety of novel systems.^73−75^ He has creatively modified the Sav host and the biotin binding pocket surrounding the metal center in a manner that tunes the catalytic properties of the ArMs. His work has demonstrated how protein-induced local environments around metallocofactors can greatly influence the function, reminiscent of directed evolution previously developed by Arnold.^21,24^ In one example, Ward and Rovis developed an ArM that catalyzed the asymmetric C–H bond functionalization reactions between pivaloyl-activated benzhydroxamic acid and acrylates with enantiometric ratios of 90:10 and overall yields of >90% (Figure 8).^76^ The ArM contains a biotinylated Rh(III)–Cp* complex (Cp* = pentamethylcyclopentadiene) as the artificial metallocofactor that was known to catalyze this reaction in the presence of acetate ion.^77−80^ The key findings were to re-engineer Sav to include two mutations—one changed a lysine to a glutamate that provided a local base in the form of a carboxylate ion near the Rh(III) center that accelerated the reaction, and another changed a serine to lysine that established the high enantioselectivity. Although no structural data were reported, the high degree of selectivity and yield supports the premise that the Rh complex is localized proximal to these two residues within a Sav subunit.

**Figure 9:**
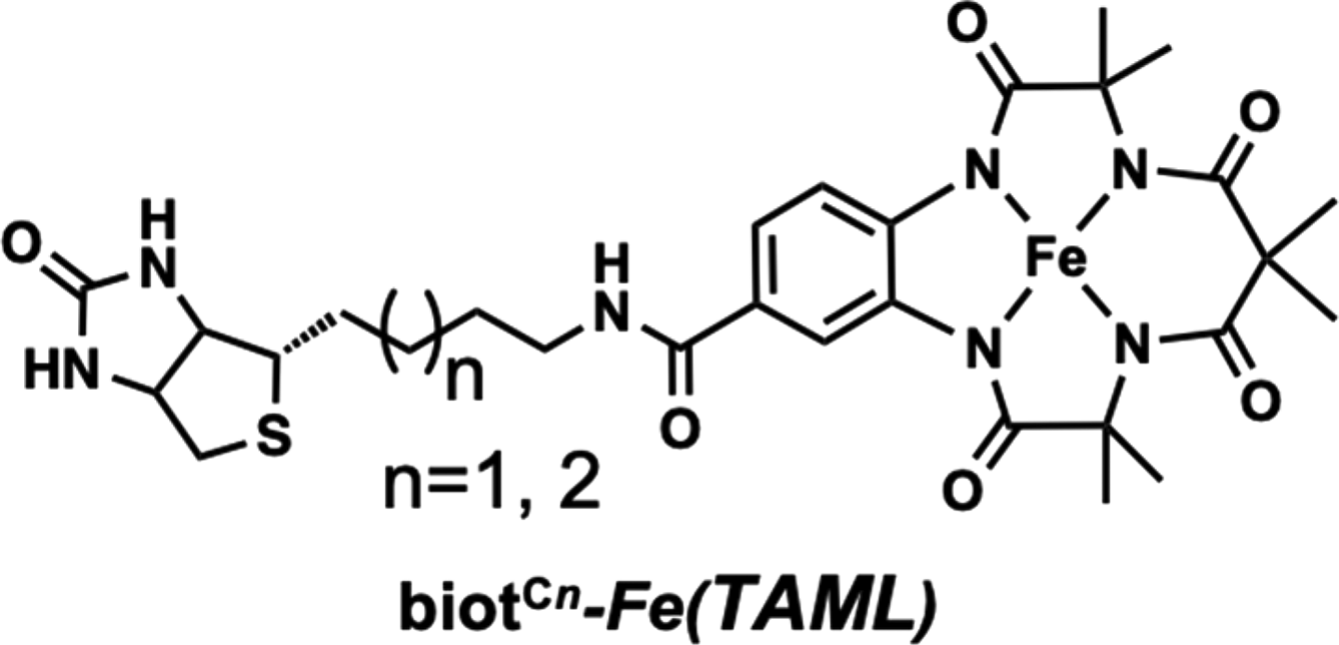
Biotinylated biot^C*n*^–Fe(TAML) complex utilized to construct an ArM in a Sav host.

Ward has since reported on several other systems that show reproducible positioning of biotinylated metallocofactors near amino acid residues within Sav variants. His method usually involves tuning the structures of both the artificial metallocofactor and the Sav host to find the optimal system. He illustrated this concept through the development of artificial hydroxylases based on Fe(TAML) complexes (TAML = tetraamido macrocyclic ligand)—these complexes were first introduced by Collins and are known to cleave C–H bonds when they are treated with H_2_O_2_.^81^ However, like the Rh systems discussed above, selectivity is harder to achieve with these types of Fe complexes. A series of ArMs with biot^C*n*^–Fe(TAML) complexes (Figure 9) were prepared with variable linker lengths between the biotin and the Fe complex.^82^ A reactivity screen was developed to monitor the hydroxylation of ethylbenzene, and an ArM prepared using a shortened biotin amine moiety linked to the Fe complex gave the highest yields and selectivity. Further reactivity studies were done using a variety of different Sav variants, and the best results were obtained with S112R and S112R/K121E Sav variants. For instance, the conversion of tetralin to tetralol was accomplished in >98% ee for the *R* isomer with 300 total turnovers.

**Figure 10:**
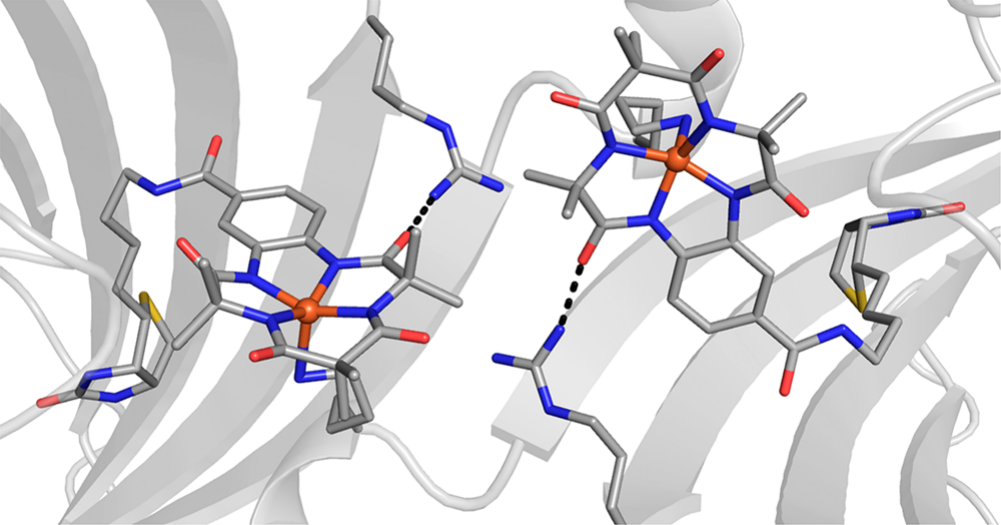
Structure of an artificial hydroxylase prepared by immobilization of a biotinylated [Fe(TAML)] complex within Sav (PDB: 6Y2M).

Companion XRD studies provided insights into structural aspects of the engineered active sites. The structure of the ArM prepared with the S112R Sav variant revealed that the Fe(TAML) moiety was locked into a specific position (100% occupancy) through interactions in both the primary and secondary coordination spheres of the Fe center. The complex was positioned such that it could form a secondary coordination sphere hydrogen bond between the arginine at 112 and a carbonyl group on the TAML ligand. This localization places the Fe center near the subunit interface of the Sav dimer and within bonding distance to K121′ (Fe–N_K121′_ = 2.3 Å), the lysine in the other subunit of the pair (Figure 10). Further studies on the ArM prepared with the S112R/K121E variant also showed a similar coordination of the carboxylate side chain from K121E′. With these findings, Ward was thus able to evaluate the key structural aspects that gave rise to observed reactivity. While the biotin provides a means to insert the artificial metallocofactor within each Sav subunit, the results point to the importance of the linker, which was necessary to localize the complex near the side chains of the amino acids at 112 and 121. It was this positioning that undoubtedly increased the influence of the Sav host on catalytic performance. Together with the reactivity results, Ward was thus able to construct an experimentally derived structure–function relationship that accurately described these engineered ArMs.

### Entatic State Models in Artificial Metalloproteins

We have been engineering ArMs with Sav hosts to emulate the active sites within metalloproteins. Our designs have used both hydrogen bonds and bonding interactions to endogenous ligands to localize the artificial metallocofactors within Sav. These bonding requirements required the embedded metal complex to be reproducibly positioned near specific amino acid side chains of Sav, which was done through a process we denoted as *positional matching*. The key aspect of this process is finding the correct linker between the biotin moiety and ligand to localize a metal complex in a specific region of the Sav subunits. Both our work and that of Ward thus established that the linker is the structural tool for positioning an artificial metallocofactor proximal to an amino acid side chain to promote a bonding interaction. Rather than use reactivity as a screen, our method takes advantage of new spectroscopic features (e.g., a new color) that are associated with coordination of an endogenous ligand to a confined metal complex. In this way, we can rapidly determine which combination of Sav variant and artificial metallocofactor gives the desired positional match.

To illustrate this concept, we describe our work on engineering entatic states within ArMs. One manner that nature has evolved to modulate the properties of metalloenzymes is through entatic states. Entatic states are uncommon geometries that result from structural restrictions placed upon the metal ion(s) and its ligands by the largely prearranged coordination environments dictated by the protein secondary structure.^83^ One proposed function of the constrained geometries of entatic states, known as the rack mechanism, is to preorient metalloenzymes toward catalytic transition states. These constrained geometries exhibited by metalloproteins are often difficult to reproduce synthetically, requiring thoughtful ligand design to mimic the steric constraints of a protein environment.

**Figure 11:**
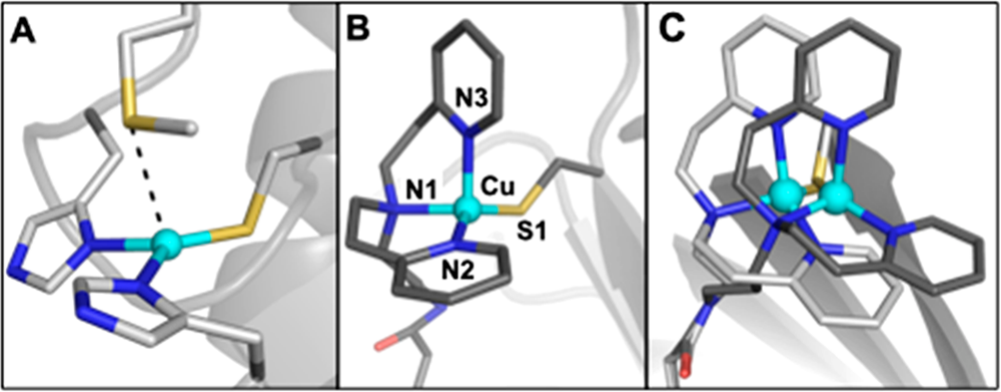
Structures of the active site in azurin (A) (PDB: 2AZA), one of the Cu ArMs that models the active site in cupredoxins (B), and an overlay of two Cu cofactors with varying linker lengths (Et (PDB: 5L3Y), Pr (PDB: 5K77)) confined within S112C Sav.

The canonical representation of entatic states in bioinorganic chemistry is the active sites of cupredoxins, commonly referred to as blue copper proteins. Cupredoxins are electron transfer proteins containing mononuclear copper sites that cycle between Cu^2+^ and Cu^+^ redox states.^84,85^ The active sites of cupredoxins exhibit a distorted-trigonal-monopyramidal coordination geometry for both Cu(I) and Cu(II) states. While it is known for Cu(I) complexes, this type of structure is rare for Cu(II) complexes because there is a strong electronic preference for the square-planar coordination geometry. The active sites within cupredoxins overcome this preference by constraining the endogenous ligands in such a manner so to force the Cu(II) state to adopt this unusual geometry (Figure 11A). These structural constraints ensure that both Cu(I) and Cu(II) states share similar coordination geometries, which promotes efficient and rapid electron transfer by adhering to the Franck–Condon principle.^86^ The trigonal plane consists of two N-histidine ligands and a cysteinyl thiolate S-donor, while the fourth axial ligand is commonly a methionine thioether S-donor but may also be derived from other amino acids. The highly covalent nature of the Cu–S_cys_ bond gives rise to the intense Sπ–Cu ligand to metal charge transfer feature that is responsible for the characteristic blue color (λ_max_ ≈ 600 nm) and the small hyperfine coupling constant observed by EPR spectroscopy (*A* ≈ 180 MHz).^86^

**Figure 12:**
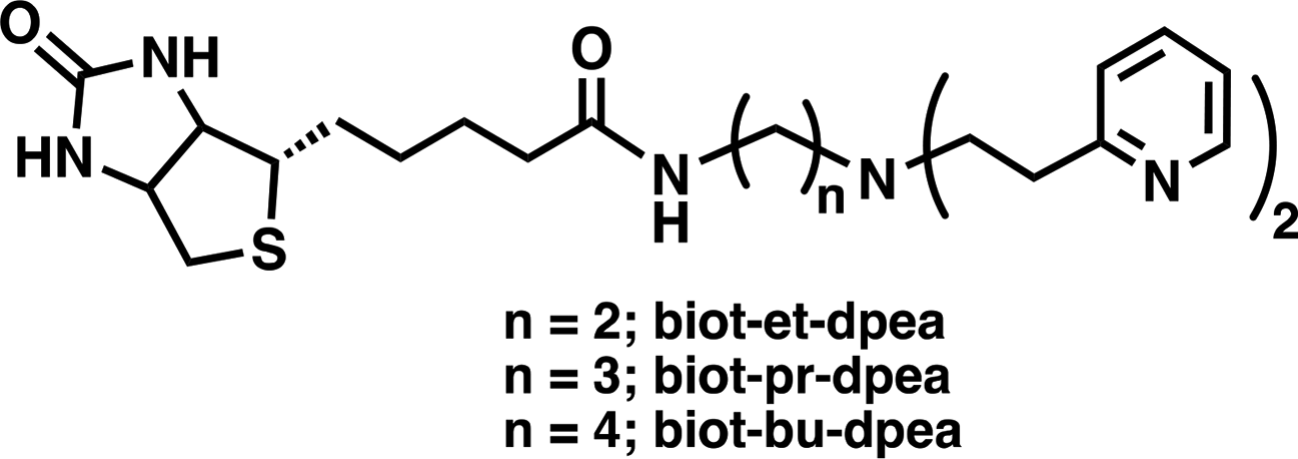
Biotinylated ligands used to prepare ArMs that model active sites within cupredoxins.

We have utilized the biotin–Sav technology to prepare artificial metalloproteins that accurately reproduce the notable structural and spectroscopic properties of native cupredoxins in their unusual Cu(II) state.^87^ A biotinylated bis[2-(2-pyridyl)ethyl]amine (dpea) was used to prepare the Cu^II^ complexes [Cu^II^(biot-n-dpea)(Cl)(H_2_O)]Cl containing a variable linker length (n = Et, Pr, Bu; Figure 12). Introduction of the synthesized Cu complexes into a S112C Sav variant resulted in a noticeable change in the optical spectra of the artificial metalloproteins with intense new charge transfer features being observable at ∼440, 570, and 700 nm and smaller hyperfine coupling constants (*A*) in the EPR spectra that were not observed in the complex alone or on introduction into WT Sav. These absorbance features are typical of blue copper electron transfer proteins such as stellacyanin and azurin and initially suggested the coordination of the cysteine thiolate residue to the synthetic Cu cofactor. The molecular structures of the artificial metalloproteins determined by high-resolution protein X-ray diffraction confirmed the thiolate coordination and revealed important structural differences in the Cu coordination sphere as a result of the ligand linker length (Figure 11B,C). For example, in comparison to the ethyl-derived Cu complex, the artificial metalloprotein derived from the propyl-linked Cu complex resulted in a shorter Cu–S bond distance (2.18 Å vs 2.11 Å) and a more trigonal monopyramidal coordination geometry akin to that of native cupredoxins (Figure 11B,C). This change in coordination at the Cu center can be directly attributed to the position of the complex in the host protein environment due to modification of the synthetic ligand linker length and is responsible for the variable intensity of absorbance features in the optical spectra in the series.

We have also applied the concept of positional matching of amino acid residues with a synthetic biotinylated iron complex to engineer a dinuclear [Fe^III^–(μ-OH)–Fe^III^] core in Sav from a mononuclear Fe complex. Dinuclear Fe cores are prevalent in biology and facilitate a range of processes including O_2_ binding, O_2_ activation, and chemical functionalization, among others. Synthetic efforts have successfully produced many examples of di-Fe^III^ cores through self-assembly of mononuclear species or dinucleating ligands containing bridging moieties. Despite the many different ligand systems that have been applied to construct dinuclear Fe^III^ cores, these complexes demonstrate only a small variation in the structural metric parameters of the di-Fe cores as a result of the highly thermodynamic nature of the bridging [Fe–(μ-O)–Fe] moiety. Counter to the norm, we have engineered ArMs that exhibit unusually long Fe···Fe distances as a result of being embedded within a protein host.

**Figure 13:**
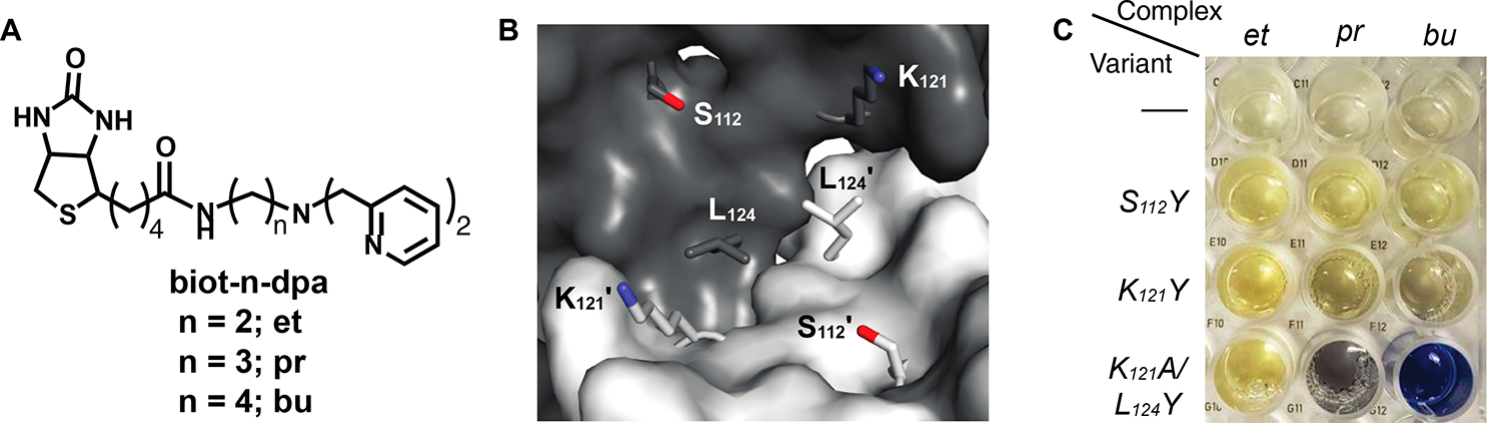
Biotinylated ligands used to prepare the Fe ArMs (A). The Sav dimer showing the amino acid residues that were mutated to Y (B). Picture of the optical assay showing the positional match that generated the di-Fe ArM (C).

Biotinylated dipyridylmethylamine (dpa) ligands containing variable ethyl, propyl, or butyl linkers were used to synthesize the Fe complexes [Fe^III^(biot-n-dpa)(OH_2_)_3_]Cl_3_ (n = Et, Pr, Bu), and a series of Sav variants (S112Y, K121Y, and K121A/L124Y) containing tyrosine mutations were selected with the intention of providing a readily visible optical assay for amino acid binding due to Fe^III^–O_Y_ interactions (Figure 13).^88^ Incubation of the complexes in the Sav variants resulted in the observation of an intense blue color (λ_max_ = 605 nm, ε_M_ = 2800 M^–1^ cm^–1^) indicative of formation of an Fe^III^–O_Y_ bond for only [Fe^III^(biot-bu-dpa)]⊂K121A/L124Y-Sav. Binding of the tyrosinate moiety to the Fe^III^ center was further supported by the observation of characteristic phenol ring vibrations and ν(Fe–O_Y_) and ν(C–O_Y_) modes for Fe^III^-bound tyrosinate species by resonance Raman spectroscopy.

The close proximity of the tyrosine residues from neighboring subunits in the assembled Sav dimer initially suggested the possibility to form bridged di-Fe cores in the vestibule. Indeed, the ⊥-mode EPR spectrum of [Fe^III^(biot-bu-dpa)]⊂K121A/L124Y-Sav was silent, suggesting magnetic coupling of the immobilized Fe centers. The Mössbauer spectrum of enriched [^57^Fe^III^(biot-bu-dpa)]⊂K121A/L124Y-Sav exhibited a single quadrupole doublet, confirming the antiferromagnetic coupling of the Fe centers. Solution X-ray absorption near edge spectroscopy (XANES) in concert with variable-temperature Mössbauer spectroscopy of enriched [^57^Fe^III^(biot-bu-dpa)]⊂K121A/L124Y-Sav crystals indicated the presence of a bridging hydroxide moiety between the two Fe centers to form a [Fe^III^–(μ-OH)–Fe^III^] core.

**Figure 14:**
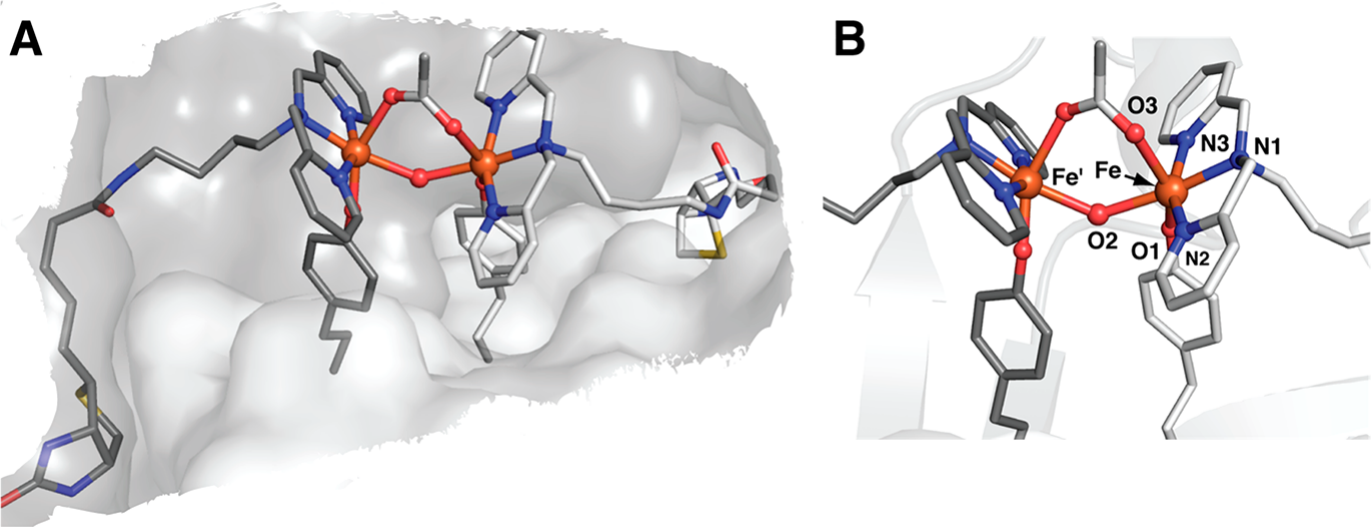
Structure of the acetate bridged di-Fe ArM in dimer of K121A/L124Y Sav (A). Close-up of the acetate bridged di-Fe site (B) (PDB: 6VO9).

The molecular structure of [Fe^III^(biot-bu-dpa)]⊂K121A/L124Y-Sav was determined by X-ray diffraction at 1.30 Å resolution (Figure 14). The molecular structure revealed a di-Fe core with six-coordinate Fe centers displaying meridional coordination of the dpa ligand and tyrosine coordination. Additional density between the two Fe centers was modeled as a disordered acetate ion and an O atom, yielding an Fe–O distance of 2.16 Å and an Fe–O–Fe bond angle of 133°. Furthermore, a long Fe···Fe′ distance of 3.96 Å was observed, in agreement with the solution measurement of 4.02 Å determined by extended X-ray fine structure (EXAFS). Molecular structures of the di-Fe ArM were also determined after the coordination of exogenous bridging acetate (OAc), azide (N_3_), and cyanide (CN) ligands. Surprisingly, the addition of exogenous ligands yields very small differences in the observed Fe···Fe′ distances with slightly contracted distances of 3.91 Å (OAc), 3.82 Å (N_3_), and 3.73 Å (CN), respectively. In comparison to both synthetic and biological precedents of [Fe^III^–(μ-OH)–Fe^III^] cores with Fe···Fe′ distances of 3.3–3.6 Å, the ArMs discussed in this series feature uncharacteristically long distances between the two Fe centers. We assert that this unusual observation is a direct result of immobilization of the complexes within the rigid Sav host. Due to both the strong affinity of Sav for biotin in concert with coordination of the Fe centers by the tyrosine amino acid residue, the Fe centers represent entatic states that are constrained in space and incapable of forming classical, more contracted di-Fe cores.

## *De Novo* Designed Artificial Metalloproteins

We have focused this Perspective on ArMs that were constructed exclusively using hosts that are from native proteins; however, there are several complementary approaches that utilize non-native protein hosts such as peptides. The most successful of these systems have predictable structures that require the peptides to fold in specific ways in order to effectively encapsulate a metallocofactor. To understand the difficulties in using this approach, it is helpful to have some context. It was once considered nearly impossible to rationally design proteins from first-principles methods (*de novo*) due to the thermodynamic properties of a protein fold. It is known that the stability of the native fold similar to than that of other conformations for a protein; thus, it is difficult to predict the overall structure of a specific sequence.^89^ However, the development of proteins from a *de novo* approach has exploded over the last 30 years through the combined efforts of several research groups and the benefits of advances in technology (e.g., solid-phase peptide synthesis and gene synthesis) and computational methods. There are several excellent reviews of *de novo* proteins,^6,90−92^ with engineered ArM being one application. Two recent examples of *de novo* ArMs are described below that illustrate aspects of the design, structure, and function.

### Encapsulated Mn Porphyrins

α-Helical bundles have long been a target for the *de novo* design of artificial metalloproteins, and numerous examples have been designed rationally and combinatorially within these scaffolds. Such designs now utilize computations and a rigorous understanding of protein folding to predict α-helical structures to atomic level precision; these predictions are further complicated by the introduction of metallocofactors. An additional challenge in the design of reactive ArMs is that they must accommodate a dynamic active site that allows substrate and cosubstrate access.

**Figure 15:**
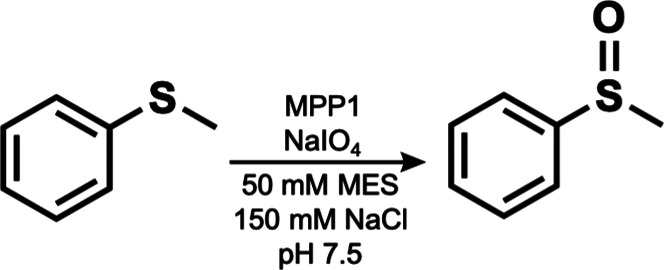
Oxidation of thioanisole catalyzed by MPP1.

Solutions to these challenges are found in the work of DeGrado, who has been a leader in the assembly of helix bundles that bind synthetic metallocofactors. In one recent report, he was successful in installing a synthetic Mn–porphyrin in a *de novo* designed four-helix bundle protein that also incorporated an access channel for external species that were necessary for catalytic function.^93^ The starting point in the development was a four-helix bundle protein (PS1) that previously had been used to bind a redox-inactive Zn–porphyrin cofactor.^94^ Computational studies on PS1 led to the new protein MPP1 (manganese porphyrin-binding protein 1), which could accommodate a larger [Mn(diphenylporphyrin)]^*n*^ (Mn(dpp)) cofactor. The computations included two additional axial ligands at the Mn center: a histidine residue to anchor the Mn(dpp) to the protein through a Mn–N bond and a coordinated dioxygen fragment. Notably, the inclusion of dioxygen in the computations ensured that the structure provided adequate space for oxidant and substrate access at an axial position of Mn(dpp). The amino acid composition was further tuned to allow flexibility to accommodate the metallocofactor and to minimize the number of oxidizable bonds near the cofactor, thus preventing undesirable oxidation of the protein backbone. The sequences of loops connecting the helices were optimized, resulting in multiple variants with stable folded cores, one of which was amenable to crystallization. Absorption and circular dichroism spectroscopy on Mn(III) MPP1 in solution supported Mn^III^(dpp) binding an axial histidine residue as designed. In further studies, it was found that Mn(III) MPP1 could be oxidized to Mn(V) MPP1 with sodium periodate. The formation was monitored by changes in the Soret band and supported by reactivity studies in which the Mn(V) MPP1 species was able to transfer an O atom to thioanisole to form a sulfoxide, as shown in Figure 15.

**Figure 16:**
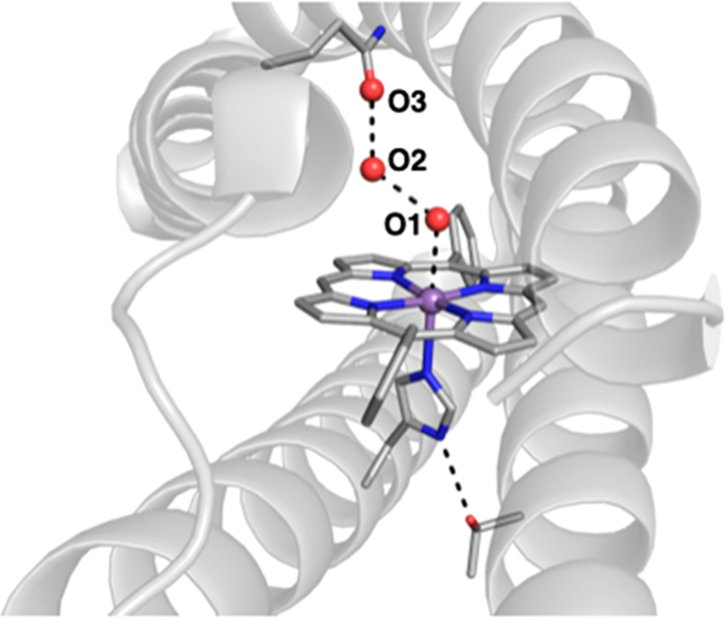
Molecular structure of the designed *de novo* ArM MPP1 (PDB: 7JRQ). Selected metrical data (Å): Mn···O1 = 2.6; O1···O2 = 2.5; O2···O3 = 2.9.

X-ray diffraction studies on single crystals of Mn(III) MPP1 verified several of the design elements of this variant that included axial ligation of the engineered histidine residue to Mn^III^(dpp) (Figure 16). Moreover, two structural waters resided in the other axial site along the vector of the dioxygen unit for which this pocket was originally designed. The waters were engaged in a H-bonding network, proposed to maintain the substrate access channel and serve as a dioxygen placeholder in the Mn(III) form of MPP1. The presence of two waters in this pocket also suggests that MPP1 can accommodate the intermediates formed during dioxygen activation. This design is not only the first crystallographically characterized *de novo* metalloporphyrin ArM but also the first to provide structural evidence for the incorporation of a substrate access channel. Such knowledge of structure–function relationships in *de novo* proteins will no doubt be critical to the strategic design of other novel ArMs.

### Active Site Mimics

DeGrado has also spearheaded the development of biomimetic active sites, and his work on di-Fe proteins has provided accurate models for several natural proteins. He again utilized four-helix bundle proteins to produce endogenous Fe binding sites housed within the interior cavity formed by the helices.^95,96^ In one example, he designed a *de novo* di-Fe protein to mimic the structural and functional properties of the metalloenzyme *p*-aminobenzoate *N*-oxygenase AurF, which catalyzes the hydroxylation of amines.^97^ A key structural feature was to increase the accessibility to the catalytic di-Fe center, which was accomplished by the mutation of several alanine residues within the interior of the protein to glycines. A second design change involved altering the primary coordination sphere around one Fe center by incorporating an additional histidine residue that coordinated to only one of the Fe centers—this new, unsymmetrical di-Fe site accurately modeled the active site in AurF and proved to be catalytic for the *N*-hydroxylation of arylamines.

Pecoraro has used *de novo* designs to prepare series of novel proteins with redox-active sites, many of which are excellent probes for electron transfer proteins. His designs utilize three-helix bundles where the metal binding site is again within the interior of the protein.^98^ He has demonstrated that these helical scaffolds may be used to model aspects of the secondary coordination spheres that surround the metallocofactors in native proteins. In order to regulate electron transfer, many proteins have evolved networks of redox active residues to enable hole hopping mechanisms to transfer electrons over large distances. Pecoraro and Aukauloo implemented this concept in a *de novo* designed protein in which a tyrosine amino acid relay in the protein scaffold mediates intramolecular electron transfer between a [Ru^III^] unit and a Fe^II^S_4_ site embedded within the protein.^99,100^

These helical structures have also proven to be sensitive scaffolds for tuning electronic properties in *de novo* designed copper proteins. We have already described the properties of the active sites in cupredoxins, the electron transfer proteins that contain Cu sites and are found in numerous organisms. The active sites within cupredoxins have evolved to control both the primary and secondary coordination spheres around the Cu center, and both are needed to ensure proper function. Modifications of either sphere can lead to differing electron transfer properties, which have been used to establish structure–function relationships. Such changes also result in pronounced changes in color and spectroscopic properties that can be used as a rapid indicator for structure–function changes. Pecoraro has used this sensitive spectroscopic feature as a probe to rationally design artificial cupredoxins that traverse a spectrum of cupredoxin properties.^101^ He designed the cupredoxin site within *de novo* three-helix bundle proteins, which takes advantage of the pseudo-*C*_3_ symmetry that is associated with this type of variant. After the first report of a red copper protein that mimics the His_2_CysGlu Cu binding site in nitrosocyanin, this *de novo* construct was redesigned to reproduce the spectroscopic properties of green and blue copper proteins as well. In one redesign, the metal binding site was rotated, which produced the green copper protein. This variant was in turn converted to a blue copper protein by *removing* an axial methionine. Additional spectroscopic studies using EPR and XAS methods provided additional evidence for the structures of these sites and the structural heterogeneity present in some variants. Nevertheless, this work exemplifies the utility of *de novo* designed proteins and allowed for a comparison of these classes of copper proteins outside of the native cupredoxin fold.

## *De Novo* Protein Assemblies

**Figure 17:**
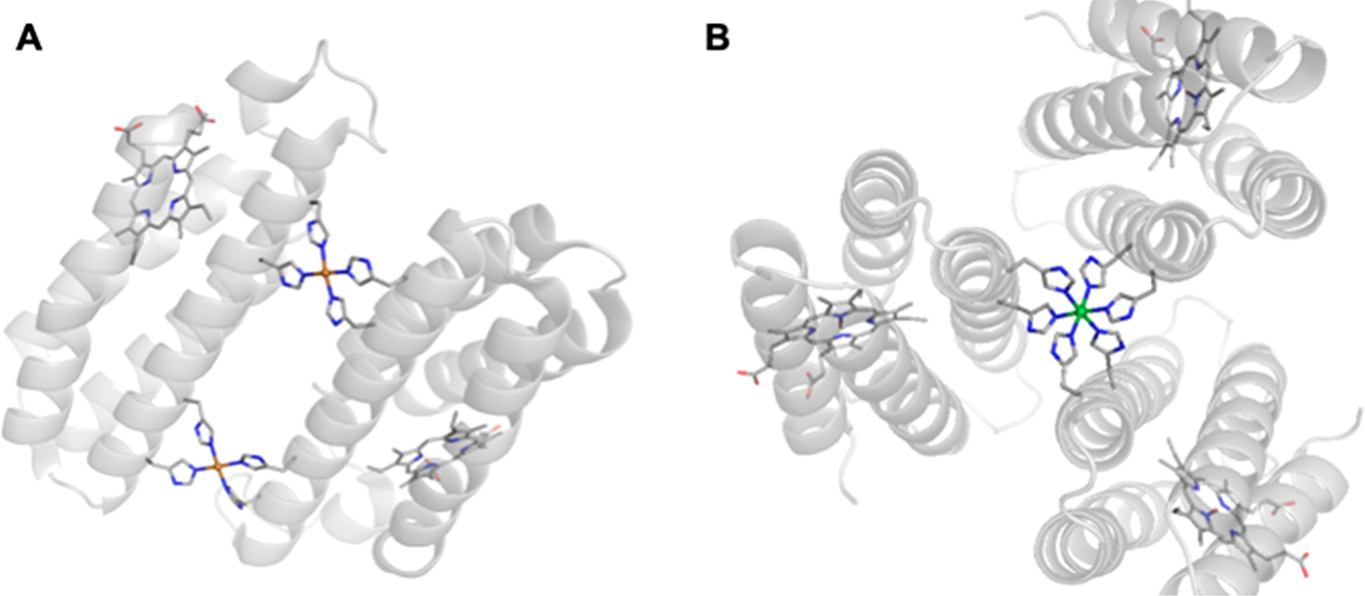
ArMs prepared using cyt *cb*562 by MDPSA depicting the different supramolecular assemblies formed in the presence of Cu (PDB: 3DE8) (A) or Ni (PDB: 3DE9) (B) metal ions.

Tezcan and co-workers have developed approaches to artificial metalloprotein design that reimagines naturally occurring proteins as ligands to form larger supramolecular assemblies. In one approach, known as metal-directed protein self-assembly (MDPSA),^102^ monomeric proteins are mutated to create new binding sites for metal ions located at the protein surface that can self-assemble into ordered oligomers upon metal binding. The monomeric heme-binding protein cyt *cb*562 was mutated to include a dihistidine motif (*i*, *i*+4) at its surface that enabled the formation of new protein oligomers upon metal coordination. The resulting oligomers were demonstrated to self-assemble into unique supramolecular arrangements depending on the preferred coordination geometry of the transition-metal ion coordinated by the engineered surface histidines (Figure 17).^103^ Following self-assembly, the protein interfaces were subjected to further mutations in order to introduce noncovalent interactions that could increase the stability of the newly formed oligomers.

**Figure 18:**
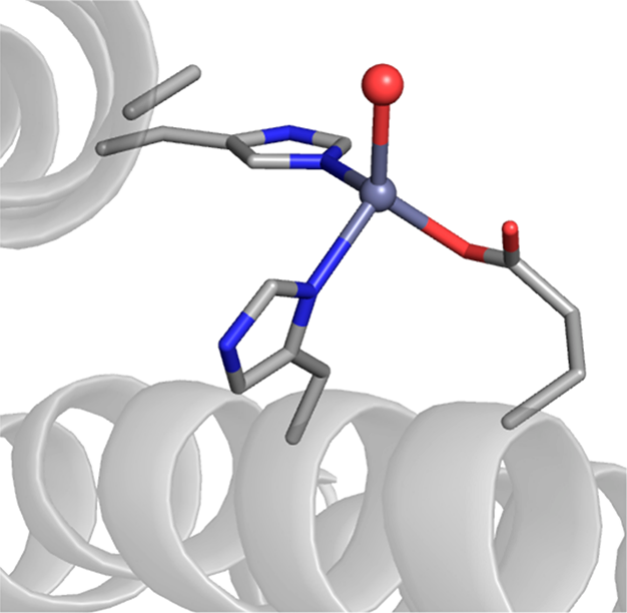
An artificial β-lactamase Zn metalloprotein using the MeTIR approach (PDB: 4U9E).

Using this metal-templated interface design (MeTIR)^104^ approach, artificial proteins could be evolved to self-assemble, even in the absence of metal ions at the protein interfaces. Notably, the designed interfaces of the artificial protein oligomers exhibited selective binding of Zn(II) (the original metal ion used in templating) in comparison to other transition-metal ions, including Cu(II), which should bind more strongly according to the Irving–Williams series.^105^ In one example, the engineered supramolecular association proved to be so favorable that the MeTIR approach could be applied to an *in vivo* assembly of the ArM. Tezcan and co-workers have since demonstrated that MeTIR can be used to design several ArMs that self-assemble into complex hierarchical structures that demonstrate properties of native metalloproteins such as preorganized metal-binding interior cavities and allostery that are difficult to achieve with the other strategies described herein. Furthermore, ArMs assembled by this approach have been evolved to acquire novel hydrolytic reactivity toward ampicillin, a β-lactam antibiotic, that functions both *in vitro* and *in vivo* (Figure 18).^106^ This result is particularly remarkable, considering that the original protein lacked any sequence or structural homology to other native β-lactamase proteins.

Tezcan and co-workers have also developed protein–metal–organic crystalline frameworks that can also be viewed as artificial metalloproteins.^107^ This approach is closely related to the aforementioned MDPSA, wherein protein surface mutations designed to coordinate a metal ion and an exogenous, metal-binding organic linker promote the assembly of 3D lattices with protein nodes. Due to the presence of the organic linker, the resulting materials do not feature the same close protein–protein interfaces as those previously described and are highly porous materials that maintain the intrinsic structural and functional properties of the incorporated protein node. Like metal–organic frameworks, the design of protein–metal–organic frameworks is highly modular, as the protein node, metal ion, and organic linker may all be varied to yield materials with new topologies.^108^ Indeed, Tezcan has shown that the thermal properties of protein–metal–organic frameworks derived from ferritin nodes are tunable and variable depending on the identity of the interstitial metal ion and organic linker.^109^

## Summary

This Perspective highlighted advances in engineering biologically relevant active sites within protein hosts. These types of ArMs have provided a wealth of information on the properties of metallocofactors and thus have proven to be useful models for natural proteins. It is striking that, within this area of research, there are several complementary methods to engineer new ArMs, and we focused on the different types of protein hosts that have been used. One common feature of these ArMs is the role that the host has in regulating the microenvironments around the metallocofactors. Protein-induced interactions, particularly those involved in noncovalent interactions, are designed into many of the active sites that have been discussed. We have previously argued that microenvironmental effects are crucial for function and are key factors in what distinguishes the functions of metalloproteins from those of synthetic systems.^110−112^ Within a larger context, ArMs offer an ideal platform to test existing ideas on how these effects regulate the properties of metal complexes that should lead to improved catalytic systems.

One idea for further development is to engineer additional sites for the specific binding of external molecules. This concept is particularly important for catalysis where selectivities and efficiencies can be amplified by designing substrate-specific binding site proximal to an artificial metallocofactor. For catalysis, the introduction of an additional catalytic site would also be advantageous for many transformations. We point to the work of Roelfes, who recently reported ArMs with dual catalytic centers.^113,114^ In one system, he introduced an ArM with two catalytic sites that acted synergistically to achieve enantioselectivities of up to 99% ee for a Michael addition reaction.^113^ The key design feature was the engineering of two different catalytic sites, in which one was comprised of an unnatural *p*-aminophenylalanine residue to activate an enal and the second contained a Lewis acidic Cu^II^ complex. The first site activated an enal, and the second activated the Michael donor by enolization and delivered it to one preferred prochiral face of the activated enal. These results show the promise of engineering multiple catalytic sites within one protein host and how increased structural complexity can be harnessed to enhance function.
